# Children, Adolescents, and Young Adults with Borderline Intellectual Functioning: Etiological, Neurophysiological, and Mri Findings in a Cohort of 651 Patients

**DOI:** 10.3390/neurolint14040080

**Published:** 2022-12-07

**Authors:** Heli Sätilä, Laura Mirjami Jolma, Mikko Koivu-Jolma

**Affiliations:** 1 Division of Child Neurology, Päijät-Häme Central Hospital, Keskussairaalankatu 7, 15850 Lahti, Finland; 2 Faculty of Medicine, University of Helsinki, Haartmaninkatu 8, P.O. Box 64, 00014 Helsinki, Finland; 3 Faculty of Science, University of Helsinki, Gustaf Hällströminkatu 2, P.O. Box 64, 00014 Helsinki, Finland

**Keywords:** borderline intellectual functioning, etiologic yield, etiological findings, MRI findings, neurophysiological findings

## Abstract

This retrospective chart review study explored the etiology, use, and yield of the etiological investigations of 651 children and adolescents diagnosed with borderline intellectual functioning (BIF). Neurological, neurodevelopmental, or neuropsychiatric comorbidities were frequent, and in 23%, the BIF diagnosis evolved into an intellectual disability (ID) by the time of discharge. A primary etiological cause was found in 37.6%, the most prevalent causes being pre- or perinatal conditions, genetic syndromes/chromosomal abnormalities, fetal exposure to maternal substance use, cerebral dysgenesis, and neurological diseases. In total, 79.1% of patients went through one or more investigations during their follow-up. The best etiologic yield leading to a diagnosis in this study population was with exome sequencing, a specific gene panel, microarrays, electroneuromyography, and brain magnetic resonance imaging (MRI). Etiological investigations were performed more frequently among those children receiving an ID diagnosis. Yet, there was no statistically significant difference in the proportion of abnormal findings between the BIF and ID groups. This may mean that the current strategy for determining the need for etiological investigations or current means to gain an etiology is still indecisive. Considering that BIF is defined to include individuals performing between normal cognitive functioning and mild ID, this implies that the prevalence would be anywhere between 7 and 14%. Thus, it could be argued whether in-depth etiological investigations may be justified in cases other than ID in this age group of children over five. With these children and adolescents, the clinicians have to discern between those with a normal variation and those having major difficulties in adaptive behavior affecting everyday life in order to specify and prescribe the rehabilitation or other measures needed. We advocate for a targeted etiological search after careful history-taking and neurological examination. National guidelines that take into account the severity of developmental delay are warranted.

## 1. Introduction

Borderline intellectual functioning (BIF) is defined as a neurodevelopmental disorder comprising individuals performing between normal cognitive functioning and mild intellectual disability (ID), defined as having an IQ test score of one to two standard deviations below average in the range of 70 to 85 and of having difficulties in adaptive behavior affecting every area of life [1,2]. In the literature, the definition of “global developmental delay” (GDD) has been reserved for children under the age of five, but some of these children may later meet the diagnostic criteria for ID [3]. In the International Classification of Diseases (ICD-10) [4], BIF has no exact definition, but the codes F83 with children or F81.3 with school-age children are used when having deficiencies in more than two domains in neurodevelopmental (language, gross and fine motor function, cognition, socioemotional, activities of daily living, memory, verbal/perceptional, attention, or executive functions) or learning functions. The Diagnostic and Statistical Manual of Mental Disorders (DSM-5) [5] suggests differentiating ID and BIF by assessing the discrepancy between cognitive and adaptive functions and not leaning solely on IQ scores. Thus, depending on the inclusion criteria, the prevalence of BIF has been estimated to vary between 7 and 14% [2,6,7,8].

The etiology of BIF is considered variable and heterogeneous. For instance, prematurity, asphyxia, intrauterine growth restriction, central nervous system malformations and infections, genetic factors and chromosomal disorders, pre-eclampsia, maternal alcohol or drug use during pregnancy, child neglect, and environmental deprivation have been recognized as causing cognitive compromise [9,10,11,12,13,14,15,16,17,18,19]. Likewise, the reported yield of different etiologic investigations is variable, ranging from 2 to 80%, depending on the developmental delay subtype, referral population, geographic and ethnic variation in the incidence of genetic developmental disorders, and use of methodology and specialized technologies [12,20,21,22,23,24,25].

The wide-ranging underlying etiology poses a challenge for clinicians with respect to the choice and extent of investigations to undertake. Recently, in child neurology and the developmental pediatric field, good practice guidelines or evidence-based recommendations for etiological evaluation have been developed [3,22,23,24,25,26]. However, these guidelines are mostly intended for investigating children under the age of five with suspected GDD or ID. Therefore, the aim of this retrospective chart review study was to explore the etiology and the use and yield of the various etiological investigations of both children and adolescents diagnosed with BIF.

## 2. Materials and Methods

This study provides a retrospective review of patient reports recorded during an 11-year period between 1 January 2010 and 31 December 2020 comprising the 651 consecutive children and adolescents (1481 appointments) who were monitored by Päijät-Häme Central Hospital’s (catchment area of 220,000 inhabitants) neuropediatric developmental unit and who had received an ICD-10 diagnosis of F83 or F81.3 by the age of 18 years or by the time of discharge. The patients’ age had to be ≥5 years at the time point the data were extracted. The data were reviewed by a single investigator (HS) from the comprehensive digital patient reports. The study was conducted according to the guidelines of the Declaration of Helsinki and approved by the local research ethics committees of Helsinki University Hospital and Päijät-Häme Central Hospital (D/2603/07.01.04.05/2019).

The data comprised the following: demographic information; age at initial evaluation and cause of referral; pre-, peri-, and postnatal birth history; maternal pregnancy history; patient and near family (first- and second-degree relatives) history; the etiological and comorbid diagnoses, (e.g., focal or generalized seizures, autistic features); and the etiological investigations conducted and results gained by the extract date (blood and/or urine, genetic, radiological, and neurophysiological tests).

Patients under school age presenting with developmental delay were evaluated by the pediatric neurologist, neuropsychologist, speech therapist, occupational therapist, and physiotherapist. The diagnosis F83 was given if there was a delay of more than 1.5 standard deviations below the mean of the norm-referenced standardized tests in two or more developmental domains. Patients attending school and having learning difficulties were evaluated by a neuropsychologist and pediatric neurologist. The diagnosis F81.3 was given for school-aged children or adolescents when two or more cognitive domains were delayed at least 1 to 2 standard deviations below the mean of the norm-referenced standardized tests, the situation did not fulfill the criteria for ID, and the child`s difficulties were not caused by a poor learning environment or a psychiatric disorder. The standardized neuropsychological tests were the Wechsler Preschool and Primary Scale of Intelligence (WPPSI-III), the Neuropsychological Test for Children’s Finnish version (NEPSY-II), and the Wechsler Intelligence Scale for Children (WISC-IV), and, in addition, the Social Responsiveness Scale (SRS-Fin), the Strengths and Difficulties Questionnaire (SDQ-Fin), and the ADHD Rating Scale-IV were used when appropriate. The tests for each participant were chosen individually according to age and profile. Moreover, as part of the diagnosis process and follow-up, information was gathered from the parents, the day care personnel, schoolteachers, and the child`s personal therapists.

During the selected inspection period, individual physicians carried out their own history collection, physical examinations, and a selection of specific investigations at their own discretion based on the suspected etiology. Thus, the exact indication for each investigation could not systematically be determined from the charts. The biochemistry tests mainly included blood count, electrolytes, glucose, lactate, venous blood gas, calcium, creatine kinase, vitamins B12 and D, and thyroid, liver, and kidney function tests. The metabolic screening comprised organic acids (urine), oligosaccharides (urine), amino acids (plasma and urine) and/or glycosaminoglycans (urine), and occasionally the creatine/guanidine acid-ratio, acylcarnitines, or sialotransferrins. Genetic testing included karyotype/microarray and/or Fragile-X or specific single gene or gene panels. Neuroimaging was mostly performed with magnetic resonance imaging (MRI; 1.5 Tesla; under sedation or anesthesia) and, on occasion, with computed tomography (CT). Neurophysiologic testing comprised electroencephalogram (EEG) and occasionally electroneuromyography (ENMG), visual evoked potential (VEP), or electroretinogram (ERG). The test results and the imaging and neurophysiological findings were categorized as normal or abnormal.

### Statistical Analysis

Descriptive statistics were generated for the whole group. Categorical variable frequencies were counted and tabulated. The mean, median, and range were calculated for numerical variables. For categorical data, the difference in proportion between the subgroups with and without an intellectual disability was tested with Fischer`s Exact Test. Statistical analysis was performed using R version 4.0. (R Core Team).

## 3. Results

A total of 651 subjects were included, of which 411 were male, and 240 were female. This corresponds to a male/female ratio of 1.7:1. The mean age at the time of data extraction was 13.7 years (with a range of 5.2 to 27). One hundred patients (15.4%) were of immigrant background. The primary reason for the referral was recorded for 552 (84.8%) cases: language delay, 274/552 (49.6%), gross motor function, 138/552 (25%), fine motor function, 58/552 (10.5%), and learning or attention difficulty, 82/552 (14.9%). The mean age when being referred to the primary care level therapist was 3.4 years (with a median of 3 and a range of 0.1 to 15), and to the multidisciplinary neurodevelopmental clinic, it was 5.4 years (with a median of 5 and a range of 0.1 to 17). At the point of data collection, the head circumference data were reported for 455 patients: 42/455 (9.2%) were microcephalic, and 16/455 (3.5%) were macrocephalic. Thirty-nine patients had problems with vision (two were totally blind), and twenty had problems with hearing.

In 149/651 (23%) cases, the BIF diagnosis evolved into an ID by the time of discharge. Neurological, neurodevelopmental, or neuropsychiatric comorbidities were frequent: 254/651 (39%) were diagnosed with attention-deficit hyperactivity disorder; 150 (23%) with an anxiety disorder or the equivalent (panic disorder and selective mutism); 104 (16%) had conduct problems; 89 (13.7%) had autistic features or autism spectrum disorder (Asperger syndrome or Asperger-like features, 74; autism, 15); 72 (11.1%) had socioemotional difficulties; 53 (8.1%) had symptoms of depression; 46 (7.1%) had Tourette, Tics, or obsessive-compulsive features; and 7 (1.1%) had psychotic symptoms. Twenty-nine (4.5%) were reported as having severe difficulties in reading comprehension and twenty-five (3.8%) in sensorimotor coordination (dyspraxia) or speech. The somatic neurological complaints were epilepsy in sixty-seven (10.3%), headache/migraine in thirty-five (5.4%), cerebral palsy/movement disorder/ataxia in twenty-one (3.2%), and pain in six (0.9%).

Overall, a primary etiological cause was found in 245/651 cases (37.6%) (Table 1). Vulnerability toward an increased cognitive-compromising risk due to maternal toxemia, gestational diabetes, epilepsy medication, smoking, or stress during pregnancy was noted with 117/651 (18%) cases. A positive family history of BIF, ID, or another neurodevelopmental or neuropsychiatric disorder was reported by 347/651 (53.3%) patients.

The number of patients whose parents reported neurological or neuropsychiatric morbidities in the first- or second-degree relatives is shown in Table 2. Genetic syndromes/chromosomal abnormalities (OR = 2.26, and *p* = 0.0048) and neurologic conditions (OR = 2.93, and *p* = 0.018) were the statistically significantly more prevalent etiological causes among those who had received the diagnosis of an ID by the time of the data extraction.

A total of 515/651 (79.1%) patients went through one or more investigations during their follow-up. The number and types of investigations performed, abnormal findings, and findings relevant to the etiology and yield of different investigations are shown in Table 3. The corresponding genetic defect was observed in forty-six family members (forty in the microarray, four in a specific single gene, and two in a karyotype).

The brain MRI was normal in 189/263 (71.9%) patients. Of the 74 abnormal MRI scans, 31 led to or affirmed an etiological diagnosis; the rest of the scans contained non-specific findings. All noted abnormal MRI findings are presented in Table 4. CT was helpful in affirming choana atresia in the CHARGE association.

A normal EEG was found in 253/382 (66.2%) patients. In four cases, an abnormal EEG led to defining the etiology (epileptic encephalopathy; two infantile spasms, one with continuous spikes and waves during sleep/CSWS, and one Rasmussen encephalitis, all without genetic findings). Most of the abnormalities noted in the EEG were due to epilepsy or other neurological conditions affecting the brain (e.g., neurofibromatosis type-1, cerebral palsy, or tuberous sclerosis) and, thus, did not add to the etiological BIF workup (Table 5). A concomitant abnormal EEG and abnormal MRI result was noted in 15 cases (only one explaining the EEG finding), a concomitant abnormal EEG and normal MRI was noted in 60 cases, and, in 17 cases, an MRI was not performed, although the EEG was abnormal.

Biochemistry tests (OR = 8.14, *p* < 0.0001), metabolic screening (OR = 5.36, *p* < 0.0001), and genetic studies (OR = 7.99, *p* < 0.0001) were performed more often among those who had received the diagnosis of an ID compared to those diagnosed with BIF. The prevalence of abnormal findings in metabolic screenings was higher in the ID group, whereas abnormal findings in the biochemistry tests and genetic studies were more common in the BIF group. The differences were not statistically significant. Likewise, EEG (OR = 4.95, *p* < 0.0001) and MRI (OR = 4.77, *p* < 0.0001) were performed more often among those who had received the diagnosis of an ID by the time of the data extraction. However, the prevalence of normal and abnormal EEGs and MRI findings did not differ significantly between the groups.

## 4. Discussion

The aim of this retrospective chart review study was to explore the etiology and the use and yield of the various etiological investigations in a cohort comprising children, adolescents, and young adults diagnosed with BIF. The data were collected from the chart database of one central hospital’s neuropediatric unit, where all the children with developmental delays or other conditions needing a specialist referral within the catchment area were sent. The patients received an ICD-10 diagnosis of F83 or F81.3 at some point during the 11-year study period; thus, the study cohort also included the cases in which the diagnosis evolved from BIF to an ID by the time of discharge. Overall, a primary etiological cause was found in 245 cases (37.6%), which concurs with the yields of 31–55% found by other etiological studies in a single pediatric neurology practice [20,21,23,27]. However, these studies have solely comprised children with global developmental delays aged under five years.

The main etiological causes identified in our study were pre- or perinatal causes and genetic syndromes/chromosomal abnormalities, followed by fetal exposure to maternal substance use, cerebral dysgenesis, and neurological conditions. This is similar to the findings of other reported series [20,21,23,27]. Categories such as maternal substance use, non-accidental or accidental brain injuries, psychosocial deprivation (13.5% of the study cases), and even some of the pre- and perinatal causes (40.4% of cases) are potentially preventable. The prevalence of maternal substance use and psychosocial deprivation was probably underdiagnosed in our cohort because access to the social service data was not available and because these issues are not often written down on the patient charts.

In our study population, the best etiological yield was gained with genetic testing: exome sequencing (all three patients; 100%), targeted specific gene tests or panels (nineteen patients; 40.4%), and microarray (forty-five patients; 23.8%). The decisions to conduct exome sequencing or a specific gene panel were made together with a medical geneticist. Moreover, in situations with uncertain microarray results, the medical geneticist was consulted. In Finland, universal national health insurance covers the medical provision; thus, the more expensive possibilities could be utilized.

In our institute, the microarray gradually substituted the G-banded karyotype from 2011 onward, and since 2014, it has been the first line investigation in genetic testing. As Fragile-X syndrome (the most common genetic disorder after Down syndrome causing ID) is not detected by microarray, the Fragile-X-test (FMR1-gene) should be remembered with patients having autistic or hyperactive behavior and dysmorphic features, such as an elongated face, broad forehead, large protruding ears, and prominent jaw. The low yield in Fragile-X in our study was a surprise as none of the 182 participants tested were carrying the mutation. This may be because of excluding the individuals already having the diagnosis of ID. However, males with clear suspicion and females with a family history of Fragile-X should be tested.

The general diagnostic yield in G-banded karyotype studies has been 4–18.6%, in Fragile-X-test, 2% [22], in microarray, 6.4–30.8% [22,28], and in exome sequencing, 31–53% [29], regardless of the severity of the developmental delay. A positive family history, the presence of syndromic or dysmorphic features, and a diagnosis of an ID are widely regarded as increasing the etiological gain [22,28]. Unfortunately, the dysmorphia in the charts in this study was not systematically described, thus preventing the further analysis of predicting factors. Analyzing the subgroups with an ID or BIF at the time of discharge revealed a significant difference in the frequency of performing different etiological testing; however, there was no statistically significant difference in the proportion of abnormal findings between the two groups. This may mean that the current strategy for determining the need for etiological investigations or current means to gain an etiology is still indecisive.

The yield of 11.8% in the brain MRI was lower than expected. The indications for the MRI were not always clearly stated in the charts, but many of the neuroimaging investigations in our study appeared to be performed because of abnormal EEG findings. However, all three tuberous sclerosis patients, 3/5 neurofibromatosis type-1 patients, and 11/16 with motor disorders (17/31; 55%) had abnormal MRI findings contributing to the etiology. Studies on neuroimaging, including CT but mostly MRI of the brain, evaluated the yield in both selectively and non-selectively performed investigations [20,24,27,30]. The reported abnormalities ranged from 0 to 98% depending on the study population, and the overall diagnostic yields ranged between 7.9 and 43.7% [20,24,27,30]. The yield has been reported to be higher (43.7% vs. 20.8%) when used with patients with abnormal neurological findings, such as motor deficits/delay, epilepsy, rapid change in head circumference, microcephaly, visual or hearing impairment, or cranial nerve abnormalities [20,27]. Our results concur with these findings.

MRI abnormalities are frequently not sufficient to determine the underlying etiology, although they are useful in the process of diagnosis; the distinction between these two factors is not always established in the studies when evaluating the factual yield [24]. The value of a negative result in leading to a diagnosis has not been established either [24]. Performing an MRI on young patients requires sedation or general anesthesia, which poses its own risks. Additionally, MRI provides incidental unexpected findings not related to the etiology or diagnosis, which may lead to additional stress in the family, further specialist referrals, and repeated follow-up investigations [31]. In our study, in nine out of forty-three incidental findings (arachnoid cysts, tuber cinereum lipoma, and pineal cysts), further referral to a neurosurgeon and follow-up imaging was required.

Studies on the diagnostic yield of routine metabolic tests in children are scarce, quoting the yield ranging from 0.25% to 5% even in the era of newborn screening [22,24,32,33]. However, the potential for finding treatable metabolic conditions, especially when visible red flag markers are present (e.g., consanguinity, regression, failure-to-thrive, or episodic symptoms), validates the investigations. Furthermore, the costs for metabolic screening are quite low, although affirmation of the diagnosis with genetic testing is often needed. In our cohort, metabolic testing yields were not observed. This is probably because of the age range of the sample, as most of the symptoms in metabolic conditions manifest during the first two years of life [32]. In Finland, congenital hypothyroidism and phenylketonuria are discovered with newborn screening, and children with obvious dysmorphic features are already investigated in the neonatal period by pediatricians. The wide newborn screening was introduced in 2014 in our institution and, thus, was not used with these study patients.

The role of EEG is mostly either affirming or helpful with differential diagnoses, as uncontrolled epilepsy or epileptic encephalopathies, such as the Landau–Kleffner syndrome or continuous spikes and waves during sleep, can be associated with regression or delay in development or speech, and some metabolic or autistic conditions may be associated with seizures [3,34]. In our study, EEG abnormalities were related to epileptiform discharges in 48/129 cases, of which only 4 pointed toward an epileptic encephalopathy. The rest of the abnormal EEG findings (81/129) included non-specific bilateral spike/slow waves, diffuse beta, or a focal finding. The high yields in CT and ENMG in our study were due to the small study sample and selection of the investigations with clinical indications.

There were several limitations in this study. Firstly, the data were collected from a single neuropediatric setting; therefore, the generalization to other populations may be limited. Secondly, though collecting the data prospectively, the analysis was conducted retrospectively. Thirdly, not all the patients in the selected BIF cohort were investigated with etiologic tests, and the intensity of the diagnostic workup depended on the physician in question, albeit having general directional guidelines. Thus, incomplete clinical documentation and the data available on, e.g., dysmorphic features, abnormal status findings, and indications for tests, prevented an analysis of probable predictive factors. Additionally, the indication of the examinations is not clear, and it is difficult to know the exact sensitivities of neuroimaging and genetic testing in children with BIF. However, this may reflect common practice. In addition, data on, e.g., custody, fostering, or need for housekeeping aid or other social support was not available.

When investigating children and adolescents with BIF, comprehensive clinical assessment with history-taking and multidisciplinary examination, together with monitoring the patient´s developmental trajectory, remains core to planning investigations. Recent evidence demonstrates that early genetic testing for those with unexplained global developmental delay, regardless of the severity of the situation, should be among the first-line investigations if another more obvious cause is not present [25]. With the advancements in genomic medicine, the paradigm of investigations might be changing, as whole genome or exome sequencing is becoming more available, making it possible to skip the previously used “broad net screening tactics” to obtain better gains with quicker responses and to be able to decrease the total costs of investigations [29,35,36,37]. On the other hand, these tests produce much information that may be of uncertain significance and may not necessarily resolve the etiology, even when considering the cost. In our study, the 100% yield in exome sequencing was because of a targeted search in selected individuals after consulting the geneticists. The determination of how the identified variant correlates with the particular developmental delay or phenotype must often be verified by medical geneticists.

How severe the degree of delay should be in order to start investigations is under debate and may depend on cultural aspects or resources available. Achieving an etiological diagnosis may also be important for providing a prognosis and discussions of probable recurrence risk, providing possible treatment, rehabilitation, or support in school, and peer support and avoidance of unnecessary or potentially more invasive and expensive diagnostic tests. Considering that BIF is defined to include individuals performing between normal cognitive functioning and mild ID having an IQ test score in the range of 70 to 85 implies that the prevalence would be anywhere between 7 and 14% [1,2]. With these children and adolescents, the clinicians have to discern between those with normal variations and those having major difficulties in adaptive behavior affecting everyday life in order to specify and prescribe the rehabilitation or other measures needed.

This being said, it could be argued whether in-depth etiological investigations may be justified in any other cases than in IDs in this age group of children over five. In this study, analyzing the subgroups with IDs or BIF at the time of discharge revealed a significant difference in the frequency of performing different etiological testing but did not allow further conclusions on whether the diagnosis affected the clinicians´ decision of selecting and performing investigations. It should be noted that etiological investigations were performed more routinely among those children having an ID diagnosis. In contrast, the investigations within the group with a BIF diagnosis were conducted only if deemed necessary. Yet, there was no statistically significant difference in the proportion of abnormal findings between the two groups. At least partly, this may be due to the rarity of abnormal findings, which calls for research with a larger sample group. There also remains the possibility that since the investigations were not routinely conducted, some proportion of etiological causes were missed in the BIF cohort. However, in conclusion, we advocate for a targeted etiological search after careful history-taking and neurological examination. In children and adolescents with BIF, monitoring the patient´s developmental trajectory, e.g., repeating neuropsychological testing in order to diagnose a later ID, may point toward planning further investigations. The biochemistry screening could be conducted mostly for exclusion purposes. EEG could be performed when seizures or regression in development occur, and MRI in targeted cases of seizures or hard neurological signs. The more expensive further etiological investigations with genetic studies (microarray and exome) could be speared for those with dysmorphic or syndromic features or for those diagnosed with ID. National guidelines, which take into account both natives´ and immigrants’ genetic backgrounds and the severity of developmental delay, are warranted.

## Figures and Tables

**Table 1:** Identified etiologies by categories (*n* = 245).

Etiologies by Categories	Patients *n* (%)
**Pre- or perinatal causes:** Asphyxia, periventricular leukomalacia,	99 (40.4)
neonatal CNS infarct or bleeding, low or very low birth weight/	
small for gestational age	
**Genetic syndromes/Chromosomal abnormalities**:	76 (31.0)
NF1, TSC, Spastic paraparesis, Crouzon, CHARGE, Helsmoortel-	
Van Der Aa, Holt-Oram, Leopard, Rieger, Rubinstein–Taybi,	
Catch-22, Noonan, Sotos, Wiever, Floating–Harbor,	
Single abnormal findings in microarray, 47XXX, 47XXY (Klinefelter),	
6/14 balanced translocation, 8/17 balanced translocation,	
9p-tetrasomia mosaicism	
**Neurologic condition:** Severe epilepsy/epileptic encephalopathy,	22 (9.0)
medulloblastoma status post, Becker muscular dystrophy, myoclonus-dystonia syndrome, congenital myasthenia gravis,	
Ataxia NAS	
**Maternal substance use:** alcohol (FASD), drugs	19 (7.8)
**Cerebral dysgenesis:** polymicrogyria, corpus callosum agenesis,	
syntelencephaly, vermis hypoplasia, pontocerebellar hypoplasia,	
bilateral heterotopia	14 (5.7)
**Brain injury (Accidental or non-accidental)**	10 (4.1)
**Psychosocial deprivation**	4 (1.6)
**CNS infection:** Toxoplasmosis	1 (0.4)

CNS = central nervous system; NF1 = neurofibromatosis type-1; TSC = tuberous sclerosis complex; FASD = fetal alcohol spectrum disorder.

**Table 2:** Reported neurological or neuropsychiatric morbidities in the near relatives of 651 study patients, many having more than one of these hereditary predispositions in the family.

Reported Morbidities in Relatives	Patients *n* (%)
Global developmental delay/intellectual disability	207 (31.8)
Specific learning disorder (dyslexia, dyscalculia)	173 (26.6)
Attention-deficit hyperactivity disorder	138 (21.2)
Specific language impairment/speech delay	99 (15.2)
Emotional/Mood disorder	93 (14.3)
Neuropsychiatric disorder (Tourette, autism spectrum)	40 (6.1)
Epilepsy	10 (1.5)
Illiteracy	2 (0.3)

**Table 3:** Investigations with their yield.

	Investigations, *n*	Abnormal Result, *n*	Result Leading to BIF Etiology, *n*	Overall Etiologic Yield, %
Biochemistry screening	497	3	1	0.2
Metabolic screening	324	23 *	0	0
TORCH antibodies	19	1	1	5.3
Genetic studies:				
Karyotype	146	8	7	4.8
Fragile-X	182	0	0	0
Microarray	189	56	45	23.8
Exome sequencing	3	3	3	100
Specific gene or panel	47	20	19	40.4
EEG	382	129 **	4	1
Brain MRI	263	74	31	11.8
Spinal cord MRI	5	1	0	0
Brain CT	8	2	1	50
ENMG	5	1	1	20
VEP	8	5	0	0
ERG	6	0	0	0

* Nonspecific findings, which normalized when controlled. ** Mostly positive because of epilepsy, tuberous sclerosis, and cerebral palsy. TORCH = Toxoplasma, rubella, cytomegalovirus, and herpes simplex virus.

**Table 4:** Abnormal brain MRI findings categorized as findings leading to etiology, contributing to etiology, and not related to etiology (incidental).

Brain MRI Findings	Total, *n*	Leading to Etiology, *n*	Contributing to Etiology, *n*	Not Related to Etiology, *n*
Non-specific ventricle dilatation/ventriculomegalia	16			16
Corpus callosum hypoplasia/agenesia	9			9
PVL (+hydrocephalus)	8	8		
Non-specific white matter signals	7			7
Chiari 1	6			6
Empty sella/Hypoplastic neurohypophysis	5			5
Arachnoid cyst	5			5
Posthemorrhage/Postischemic defect	4	4		
Posttraumatic/postoperative defect	4	2	1	1
Astrosytomas, tubers, subependymal noduses related to TSC	3	3		
Non-specific enlargement of subarachnoid spaces	3			3
Hamartomas related to NF1	3	3		
Pineal cyst	3			3
Venous angioma	2			2
Syntelencephalia	2	2		
Polymicrogyria + Corpus callosum agenesia	1	1		
Pontocerebellar hypoplasia	1	1		
Medulloblastooma, status post	1	1		
Bilateral heterotopia	1	1		
Dandy–Walker	1	1		
Tuber cinereum lipoma	1			1
Cisterna magna	1			1
Papilla atrophy	1			1
Porencephalic cyst	1			1
Vermis hypoplasia	3	2	1	

PVL = periventricular leucomalasia; TSC = tuberous sclerosis complex; NF1 = neurofibromatosis type 1.

**Table 5:** Abnormal EEG findings categorized as findings leading to etiology, contributing to etiology, and not related to etiology (incidental).

EEG Findings	Total, *n*	Leading to Etiology, *n*	Contributing to Etiology, *n*	Not related to Etiology, *n*
Focal epileptiform abnormalities without epilepsy	29		2	27
Focal epileptiform abnormalities related to focal epilepsy	27	1	3	22
Diffuse beta/slow waves/abnormal background activity	26	1	1	24
Bilateral spike/slow waves without epilepsy	22		2	20
Bilateral epileptiform abnormalities related to generalized epilepsy	17	1	1	15
CSWS	4	1	3	
Focal epileptiform abnormalities related to prenatal/perinatal incident	4		4	

CSWS = Continuous spikes and waves during sleep.
